# The PROTECCT-M study: a cohort study investigating associations between novel specific biomarkers, patient-related, healthcare system markers and the trajectory of COPD patients treated in primary care

**DOI:** 10.1186/1471-2466-14-88

**Published:** 2014-05-20

**Authors:** Jens Søndergaard, Anders Halling

**Affiliations:** 1 Research Unit of General Practice, Institute of Public Health, University of Southern Denmark, J.B. Winsløws Vej 9a, Odense DK-5000, Denmark

## Abstract

**Background:**

Chronic Obstructive Pulmonary Disease (COPD) is the most common severe chronic disease in primary care. It is typically diagnosed at a late stage, and it is also difficult to predict its trajectory and hence to tailor treatment and rehabilitation. The overall aim is to study determinants of exacerbations of COPD treated in primary care and to study, if the prognosis is related to patient-related, healthcare system markers or levels of the potential biomarkers such as microfibrillar-associated protein 4 (MFAP4) and surfactant protein D (SP-D). Furthermore, we aim to establish a cohort of COPD patients treated in Danish primary care comprising register data, data captured from the GPs’ electronic patient record system (EPR) and a biobank in order to make analyses on factors associated with different tractories of COPD treated in primary care.

**Methods/design:**

A cohort study of incident and prevalent COPD patients treated and followed by their GPs using data capture, which is a computer system collecting data from the GPs’ own EPR and transferring them to the Danish General Practice Research Database. The participating COPD patients were investigated at a baseline consultation by their own GP, and the results were registered using a pop-up menu by the GP. During the consultation blood samples were taken and the patients were given a questionnaire. The patients will then be followed prospectively at yearly consultations and in between these consultations by means of the data capture system. The collected data will also be combined with register data from other sources. The data collection started in December 2012, and so far 30 practices with 77 GPs have included about 350 patients. The study aims to include 2000 patients till the end of 2016, and after that to continue to collect data on these patients using the data capture system.

**Discussion:**

The GP currently lacks tools to predict trajectory of their COPD patients. The measurement of lung function only reflects loss of lung capacity and not disease activity. Use of biomarkers for detection of early COPD could be a possible way of predicting trajectory to aid both the GP and his/her patients. This study aims to provide evidence of determinants of a COPD trajectory, including novel specific biomarkers and other patient- and healthcare system-related markers.

**Trial registration:**

ClinicalTrials.gov Protocol Registration System, Identifier: NCT01698151

## Background

Chronic obstructive pulmonary disease (COPD) is a common chronic, often life-threatening disease characterised by a permanent and progressive airway obstruction. COPD is clinically defined as having irreversibly reduced airway function of an obstructive type (FEV_1_/FVC <70%) [1-3]. According to the World Health Organisation 80 million people suffer from moderate to severe COPD worldwide, and it is expected that this will increase to become the third most important cause of death. It is estimated that in the population of 5.5 million inhabitants in Denmark, 430 000 people suffer from COPD, 230 000 of these from moderate COPD and 40 000 from severe COPD [4]. Denmark has one of the highest prevalences and mortality rates of COPD, probably primarily due to frequent tobacco use. Hospital visits for this group of patients are common and account for a substantial part of healthcare costs as a whole [5,6]. Some 85% of patients with COPD are or have been smokers, and it is estimated that 35-40% of smokers develop the disease [7]. COPD is associated with a low socioeconomic status, markedly reduced quality of life, large use of prescribed drugs and frequent contacts with the healthcare system [2,6,8]. COPD is a progressive disease developing over decades, but if the degree of disease progression among COPD patients is not stopped, in 10-15 years there will be an even greater need for hospital beds, medical treatment and rehabilitation at huge expenditure.

In Denmark 98% of the population is listed with a GP who provides primary care for a defined part of the population. A large part of the population consults their GP several times during a year. Most COPD patients are diagnosed by their GP, typically after a long period of productive cough and reduced function in daily life with dyspnoea. The maximum prevalence is reached at about the age of 80 years. Today much of a patient´s lung function is already lost at the time of diagnosis. Despite massive preventive measures COPD is a major and rising problem in Denmark [9]. The diagnosis is made by investigation of lung function (spirometry), which can be performed by the GP, but it takes time and requires skill and is therefore not always performed [10,11]. The measurement of lung function can, however, neither in light or moderate COPD predict the development of the disease, as it only reflects loss of lung capacity and not disease activity. Exacerbations are associated with significant mortality, loss in lung function and deterioration in quality of life [12-14]. Apart from previous exacerbation history, we currently have no good tools to identify frequent exacerbators. Use of biomarkers for detection of early COPD could be a possible new way [15,16] of predicting prognosis to aid both the GP and his/her patients, but until now no biomarkers of disease activity have been shown to predict a COPD trajectory. However, through an ongoing collaboration [17] we have established a working relationship with specialists in molecular medicine. Two molecules have especially been in focus for their studies: surfactant protein D (SP-D) [18,19] and microfibrillar-associated protein 4 (MFAP4). Lack of either SP-D or MFAP4 leads to development of emphysema in mice and could thus be involved in the heterogeneity in disease development and progression of COPD. Both molecules are primarily expressed in the lungs, and both molecules are present in humans. In the ECLIPSE cohort SP-D has been shown to be associated with exacerbations, but not with the severity of COPD [18].

### Objective

The overall aim is to establish a cohort of COPD patients in primary care and to study determinants of COPD exacerbations related to the patient and the healthcare system. The aim is also to test the ability of MFAP4 and SP-D as biomarkers to predict exacerbations, so that treatment and rehabilitation efforts can be focused on the patient groups that benefit the most.

## Methods and design

### Participants

Patients with a diagnosis of COPD (ICPC code R95-) are eligible for participation, if they are listed with a GP participating in the study, are aged ≥ 40 years, understand Danish, do not have severe psychiatric or cognitive disease and are able to visit the GP surgery. Participating GPs are also encouraged to screen for COPD in patients with relevant symptoms or risk factors, and these patients are then offered to participate in the study if a diagnosis is made.

**Figure 1:**
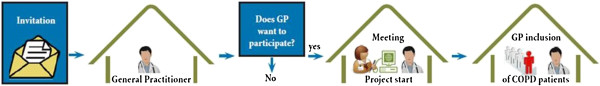
**Schematic diagram of study.** General practitioners working on Funen (500 000 inhabitants) were invited by the study nurse to participate in the PROTECCT-M study if they were using data capture (Figure 2).

**Figure 2:**
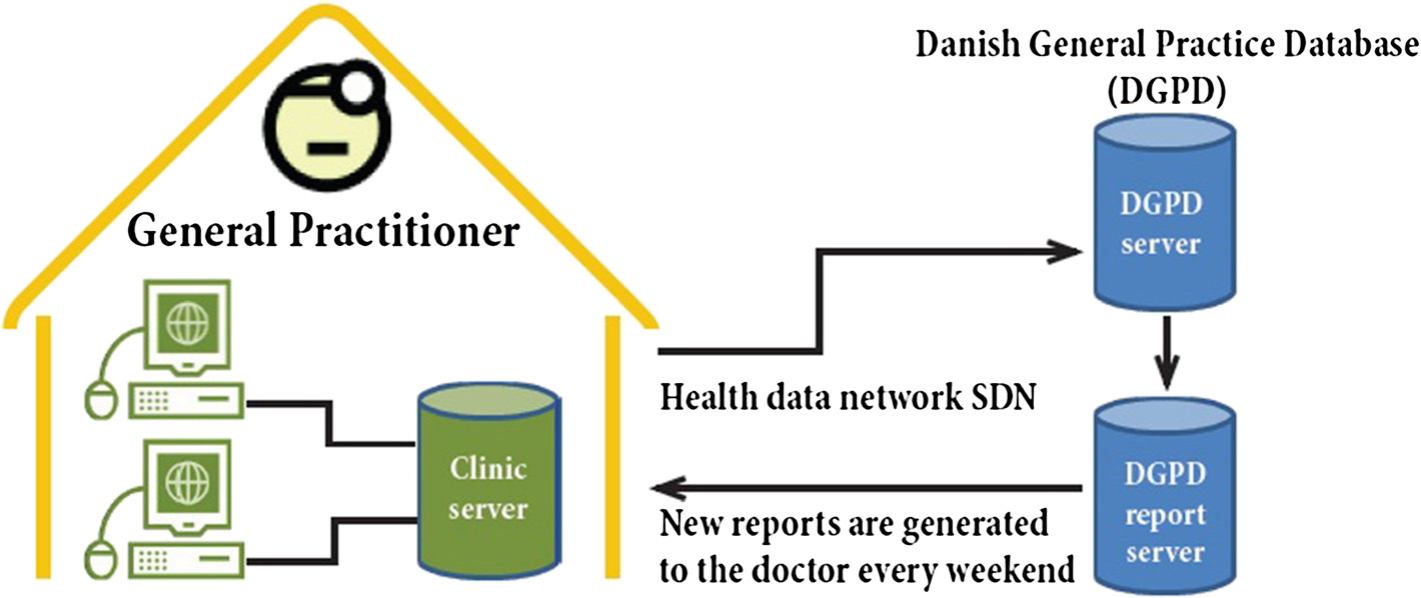
**Data capture.** The Sentinel Data Capture program collects key data as it is entered into the GP´s electronic patient record system. The collected data are prescribed drugs, National Health Service disbursement codes, laboratory analysis results and ICPC diagnoses. In addition, data are collected via pop-up menu for specific diseases or conditions. Every night data are sent to the Danish General Practice Research Database (DGPD), through the Sentinel Data Network (SDN). Treatment quality reports are generated every weekend and can be accessed by the GPs.

**Figure 3:**
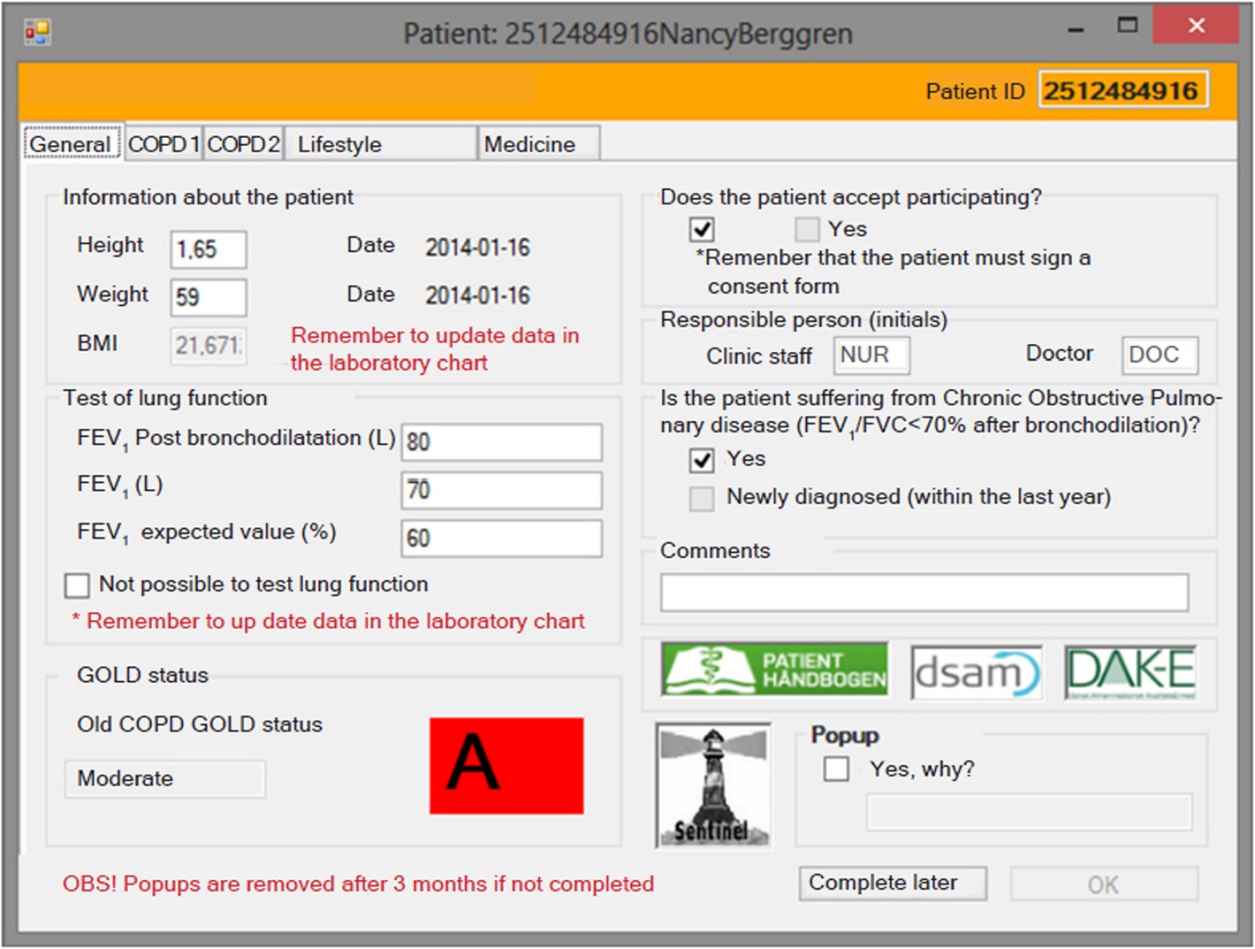
**Data collection.** The PROTECCT-M COPD pop-up is displayed at the baseline visit and then once a year, when the GP enters an ICPC diagnosis for COPD (R95-) in his electronic patient record system. The data is given as an example and is not from a real patient.

All GPs on the island of Funen using data capture [20] in the spring of 2012 were invited to participate. Invitations, startup meetings and monitoring of participating GPs were handled by one study nurse (Figure 1). At the startup meeting the participating GPs were given a binder containing a standard operating procedure, including protocols, questionnaires etc. Data capture is an electronic system that captures data from the electronic patient records (EPR) of participating GPs and transfers them to the Danish General Practice Database (Figure 2). Data collected automatically comprise all drug prescriptions, all diagnoses of patient contacts, all disbursement codes and all laboratory data recorded in the patient file. On the day of the inclusion a pop-up will appear (Figure 3) and subsequently once a year, where the GP fills in questions about COPD care. The participating GPs are also reminded to reinvestigate COPD patients included by the project nurse. The data from the fields in the pop-up are also stored in the laboratory data.

The first practice to include patients started in October 2012. Since 2013 it is mandatory for Danish GPs to participate in data capture. New practices starting to use data capture have been continuingly invited to participate by the study nurse. Currently (January 2014) 30 practices with 77 GPs and 350 COPD patients have been recruited. The study is planned to continue to include patients till the end of 2016, but will continue to collect data on the included patients through the data capture system after the project period.

### Study design

The PROTECCT-M study (Prognosis, Treatment and Course of Events of COPD and the Use of BioMarkers) is a prospective observational cohort study in primary care on the island of Funen (approx. 500 000 inhabitants) in Denmark. The decision to restrict the study to Funen was made in order to secure quality of transport of blood samples to Odense University Hospital.

The study is the primary care part of a collaborative partnership with researchers interested in COPD at the University of Southern Denmark, comprising researchers also in molecular medicine, lung medicine and health economics [17]. The group has a common funding from the Danish Strategic Research Fund.

After the patient has been informed about the study and signed an informed consent to participate, he/she is invited to their usual GP for a baseline consultation, where a pop-up menu in the EPR is filled in by the GP. This pop-up window is triggered by the ICPC R95 diagnosis (Figure 3). The information collected by means of the pop-up consists of all relevant indicators of the patient’s COPD disease status, as recommended by the Danish College of General Practitioners, and information concerning lifestyle. During these consultations blood samples are collected and transferred to Odense University Hospital, where the specimens are prepared and then stored at the OPEN biobank [21] until analysis.

After the baseline consultation the patients are given a questionnaire (including respiratory, quality of life and activity of daily living questions), which they are asked to fill in and send to the Research Unit of General Practice using an attached prepaid envelope.

The participating GPs are encouraged to reinvestigate included patients at least yearly after the baseline consultation, and they get reminders both from the data capture system and the research nurse. The data collected during the yearly visits and also between visits are transferred automatically by the data capture system and stored in the Danish General Practice Research Database.

For specific research questions data will also be obtained from Statistics Denmark, Odense Pharmacoepidemiological Database (OPED), the National Patient Registry (Landspatientregistret), the Danish Health Insurance Registry and others.

Through the CEKOL collaboration [17] the blood samples collected during the baseline visits will be analysed for SP-D and MFAP4 levels at the Institute of Medical Biology, University of Southern Denmark, using the funding already obtained.

### Time schedule

All permissions are in place. The inclusion of patients is ongoing. The current project is planned to continue including patients till the end of 2016. Using the data capture technology patient data can be collected continuously from the specially developed COPD pop-up window and the GP´s electronic patient record system after the project period.

### Outcomes

The primary patient outcome is number of exacerbations. The number of exacerbations during the preceding year requiring antibiotic treatment or hospitalisation was recorded in the pop-up menu (Figure 3). The definition of an exacerbation could be based on this or based on prescribed treatment of antibiotics and/or corticosteroids alone or in combination based on registers on prescribed medication or hospitalisations due to infection or COPD.

### Determinants of exacerbations

#### ***Demographic and clinical characteristics***

Age

Gender

Comorbidity

Body mass index

Smoking status

Exacerbations during the preceding year

MRC, Medical Research Council Breathlessness Scale [22]

Educational level

#### ***Lung function***

FEV1 – per 100 ml decrease

FEV1 – per 5% decrease in % of predicted value

GOLD stage – per increase to next stage

FEV1: FVC per 1% decrease

FVC – per 100 ml decrease

#### ***Patient-reported outcomes***

CCQ, clinical COPD questionnaire [23]

SGRQ, St George Respiratory Questionnaire [24,25]

ADL, Activity of Daily Living, [26,27]

MDI, Major Depression Inventory [28]

MFI - 20, Multidimensional Fatigue Inventory [29,30]

Questions about lifestyle (smoking habits, dietary habits, exercise, alcohol consumption) [31]

SF-12 [32]

EQ5D [33]

#### ***Biomarkers***

Surfactant protein D (SP-D) [18,19]

Microfibrillar-associated protein 4 (MFAP4)

### Data analysis

Descriptive statistics will be generated for all variables. Multivariate logistic regression models will be used to determine variables independently associated with the outcome variable. An analysis will be considered statistically significant at a probability level of p < 0.05 based on two-sided tests.

## Discussion

COPD is a common disease in the population, which is often diagnosed and followed in primary care in the early phases. COPD is a complex disease with both pulmonary and extrapulmonary manifestations. Some patients deteriorate quickly, while others remain stable for a long period. More accurate tools/markers, which can predict prognosis and thus identify groups of patients in need of specific therapies or rehabilitation are needed. COPD can be a challenge to the GP since there are few tools to elucidate the prognosis of the individual patient. For such tools to be of “clinical utility” [34] it is of importance that the evidence is generated in general practice under conditions that resemble normal clinical practice as closely as possible, which we believe that data collection through data capture offers. It is thus of importance that GPs are interested in participating in studies such as the present study aiming at creating more personalised medicine for COPD patients treated in primary care. The aim was to study factors determining frequency of exacerbations and to investigate if the biomarkers SP-D and MFAP4 have such a role in COPD patients treated in primary care.

### Ethical aspects

The study will be conducted in accordance with the Helsinki declaration. The study has been approved by the Regional Scientific Ethics Committee in the Region of Southern Denmark, project id: S20110163. The Danish Data Protection Agency has been notified and has approved the study. The study has also been registered at ClinicalTrials.gov, Identifier: NCT01698151.

## Competing interests

JS is a member of an advisory board for the Boehringer Ingelheim Company. AH declares no competing interests.

## Authors’ contributions

AH and JS conceived and designed the study. AH wrote the first draft of the manuscript and JS provided constructive opinions and suggestions. AH has been principal investigator for the study. JS wrote the application that received funding for the study. Both authors have read and approved the final version of the study.

## Pre-publication history

The pre-publication history for this paper can be accessed here:

http://www.biomedcentral.com/1471-2466/14/88/prepub
